# Preliminary characterization of the skin microbiota in basal cell carcinoma: An exploratory pilot study in Korean patients

**DOI:** 10.71150/jm.2511012

**Published:** 2026-02-13

**Authors:** Hye Lim Keum, Woo Jun Sul, Suyeon Kim, In-Young Chung, Ara Koh, Hei Sung Kim

**Affiliations:** 1 Systems Microbial Ecology Laboratory, Department of Systems Biotechnology, Chung-Ang University, Anseong 17546, Republic of Korea; 2 Department of Dermatology, Incheon St. Mary’s Hospital, The Catholic University of Korea, Seoul 06591, Republic of Korea; 3 Department of Life Sciences, POSTECH, Pohang 37673, Republic of Korea

**Keywords:** skin microbiota, basal cell carcinoma, Korean

## Abstract

Basal cell carcinoma (BCC) is the most common form of skin cancer, with ultraviolet radiation recognized as the primary environmental driver; however, the potential contribution of alterations in the skin microbiota remains incompletely understood, particularly in Asian populations. This exploratory pilot study describes bacterial community patterns in BCC lesions compared with contralateral clinically normal skin in 20 Korean patients. Lesional and contralateral samples were obtained using paired skin swabs and punch biopsies and analyzed by full-length 16S rRNA gene sequencing, with targeted quantitative PCR (qPCR) of the *roxP* antioxidant gene of *Cutibacterium acnes*. Given the low-biomass nature of skin samples and the exploratory design, analyses focused on descriptive trends rather than confirmatory inference. Across available samples, *C. acnes* was the dominant taxon, with a trend toward lower relative abundance in BCC lesions, particularly in biopsy-derived datasets. Microbial evenness appeared higher in lesions than controls. Predictive functional profiling suggested reduced representation of vitamin B6 metabolism pathways in lesions, while qPCR analysis of swab samples showed a trend toward lower *roxP*/16S rRNA ratios in BCC-associated microbiota. These findings should be interpreted cautiously in light of methodological constraints, including sample heterogeneity, lidocaine exposure prior to biopsy, absence of sequencing-based negative controls, and reliance on predictive functional inference. Overall, this pilot study highlights potential differences in skin bacterial community structure between BCC lesions and contralateral skin in a Korean cohort. Larger, methodologically optimized studies incorporating metagenomic and functional validation will be required to determine whether these microbiota shifts contribute to, or result from, BCC-associated changes in the cutaneous environment.

## Introduction

Basal cell carcinoma (BCC) is the most common malignancy of the skin and accounts for the majority of non-melanoma skin cancers worldwide. Although BCC rarely metastasizes, it can cause substantial local tissue destruction and morbidity. Ultraviolet radiation (UVR) is the primary environmental risk factor for BCC, inducing DNA damage, oxidative stress, and local immune dysregulation. However, BCC development is increasingly recognized as a multifactorial process influenced by host genetics, immune responses, and the local cutaneous microenvironment (Woo et al., 2022).

The skin microbiota plays an important role in maintaining cutaneous homeostasis through modulation of immune signaling, barrier integrity, and oxidative balance (Byrd et al., 2018; Woo and Kim, 2024). Alterations in bacterial community structure have been implicated in inflammatory skin disorders and, more recently, in skin carcinogenesis and UV-induced immune modulation (Nakatsuji et al., 2021; Patra et al., 2019). Experimental and clinical studies have demonstrated that UV exposure itself can perturb skin microbial composition and host–microbe interactions, supporting a mechanistic link between chronic photodamage and microbiota alteration (Burns et al., 2019; Grant et al., 2024; Patra et al., 2019).

In keratinocyte cancers and their precursors, emerging evidence suggests lesion-associated microbial shifts relative to surrounding skin. Microbiota differences have been reported in actinic keratosis and cutaneous squamous cell carcinoma, conditions that share cumulative UV exposure and field cancerization with BCC. These studies describe altered community composition, reduced dominance of sebaceous-site commensals, and increased microbial evenness in lesional or photodamaged skin, although findings vary across cohorts and methodologies (Krueger et al., 2022; Voigt et al., 2022; Wood et al., 2018). Despite growing interest in the role of the skin microbiota in non-melanoma skin cancers, most available data derive from Western populations, and studies specifically addressing BCC remain limited (Kim and Kim, 2019; Kim et al., 2023; Lee et al., 2019).

Among dominant skin commensals, *Cutibacterium acnes* has been proposed to contribute to oxidative homeostasis through multiple antioxidant systems, including the secreted protein RoxP. RoxP has been shown to neutralize extracellular reactive oxygen species in vitro, although its in vivo relevance relative to other bacterial antioxidant pathways remains incompletely defined (Allhorn et al., 2016; Cros et al., 2023). Whether changes in *C. acnes* abundance or antioxidant potential are associated with BCC remains unclear.

The present study was designed as an exploratory pilot investigation to describe bacterial community patterns in BCC lesions compared with contralateral clinically normal skin in Korean patients. Using paired swab and biopsy sampling with full-length 16S rRNA sequencing and targeted *roxP* quantification, we aimed to identify preliminary trends that may inform hypotheses for future mechanistic studies. Given the modest cohort size and methodological constraints inherent to low-biomass skin microbiota research, all findings are interpreted as descriptive rather than causal.

## Materials and Methods

### Study design and participants

This pilot study was conducted at the Department of Dermatology, Incheon St. Mary’s Hospital, The Catholic University of Korea, between July 2021 and December 2022. Twenty Korean patients with histologically confirmed BCC were enrolled after providing written informed consent (Table 1). Exclusion criteria included systemic antibiotic use within four weeks or topical antibiotic application within two weeks prior to sampling. The study was approved by the institutional ethics committee (OC21TISI0038).

### Sampling procedures

For each participant, microbiota samples were collected from the BCC lesion and from contralateral clinically normal skin at an anatomically matched site whenever possible. Two sampling approaches were used: surface swabbing and punch biopsy.

Swab sampling was performed first using sterile flocked swabs pre-moistened with buffer (0.15 mol/L NaCl with 0.1% Tween 20), brushed 20 times across the target site in a crosswise direction. Punch biopsies (3 mm) were subsequently obtained after local anesthesia with 2% lidocaine; no antiseptic agents were applied prior to sampling. Lidocaine has documented antimicrobial activity and may influence biopsy-derived microbiota profiles; lesion and control sites were handled identically within each patient.

Samples were immediately placed in sterile tubes, stored at −80°C, and processed within six months. Prolonged frozen storage may affect DNA integrity, particularly for Gram-positive bacteria. Not all participants contributed both swab and biopsy samples, resulting in incomplete pairing for some analyses.

An independently processed air swab blank sequenced using a MiSeq V4–V5 workflow yielded 916 total reads. Detected taxa are provided for qualitative reference only and were not used for read subtraction or statistical decontamination due to differences in sequencing platform and amplicon region.

### DNA extraction and sequencing

DNA was extracted using the DNeasy PowerSoil Pro Kit (Qiagen, Germany), which is optimized for environmental samples but may show reduced lysis efficiency for certain Gram-positive taxa, such as *C. acnes*. Negative extraction or PCR blanks were not sequenced for the PacBio dataset, precluding formal contaminant filtering.

Full-length 16S rRNA genes were amplified using primers 27F and 1492R and sequenced on the PacBio Sequel II platform. Bioinformatic processing was performed using QIIME2 with DADA2 for denoising and amplicon sequence variant (ASV) inference. Taxonomic assignment was conducted using the RDP database (trainset 18) (Bolyen et al., 2019; Callahan et al., 2016; Cole et al., 2009; Martin, 2011). Analyses were limited to species-level resolution.

### Functional prediction

Predictive functional profiling was performed using PICRUSt2 to estimate KEGG pathway representation. Given the use of 16S rRNA amplicon data derived from low-biomass skin samples and incomplete reference genome representation of skin commensals, all functional predictions were considered exploratory and interpreted as inferred genomic potential rather than measured biological activity.

### Quantification of *roxP* gene

Absolute quantification of *roxP* gene was performed by qPCR in swab samples with sufficient DNA yield (Andersson et al., 2019). Primer efficiencies and standard curves were validated prior to analysis, and *roxP* copy number was normalized to total bacterial 16S rRNA gene copy number. Each reaction was performed in technical duplicates. Primer specificity was validated in silico against RefSeq bacterial genomes and the NCBI nt database.

### Statistical analysis

Statistical analyses were conducted in R (v4.1.2) and GraphPad Prism (v8.4.3). Paired statistical tests were used whenever complete lesion–control pairs were available; unpaired tests were applied only when pairing was incomplete. Alpha diversity metrics were compared using paired t-tests or Wilcoxon signed-rank tests as appropriate.

Beta diversity was assessed using PERMANOVA (adonis2, vegan), with subject ID included as a blocking factor when applicable. Because ANOSIM does not account for paired sampling, beta-diversity results are interpreted descriptively. PICRUSt-based functional predictions were compared using paired Wilcoxon signed-rank tests with Benjamini–Hochberg correction. Multivariate analyses, including Random Forest feature ranking and SparCC network inference, were considered exploratory and hypothesis-generating.

## Results

### Study cohort and sample overview

The cohort included 20 patients (12 female, 8 male; mean age 79.0 years, range 53–100). Most lesions were located on the face, head, or neck, with one perianal lesion (Table 1). Comorbidities such as diabetes mellitus, hypertension, chronic kidney disease, and dyslipidemia were common and are acknowledged as potential confounders.

A total of 15 biopsy pairs and 13 swab pairs were available for analysis.

### Microbial composition and diversity

**Fig. 1.**
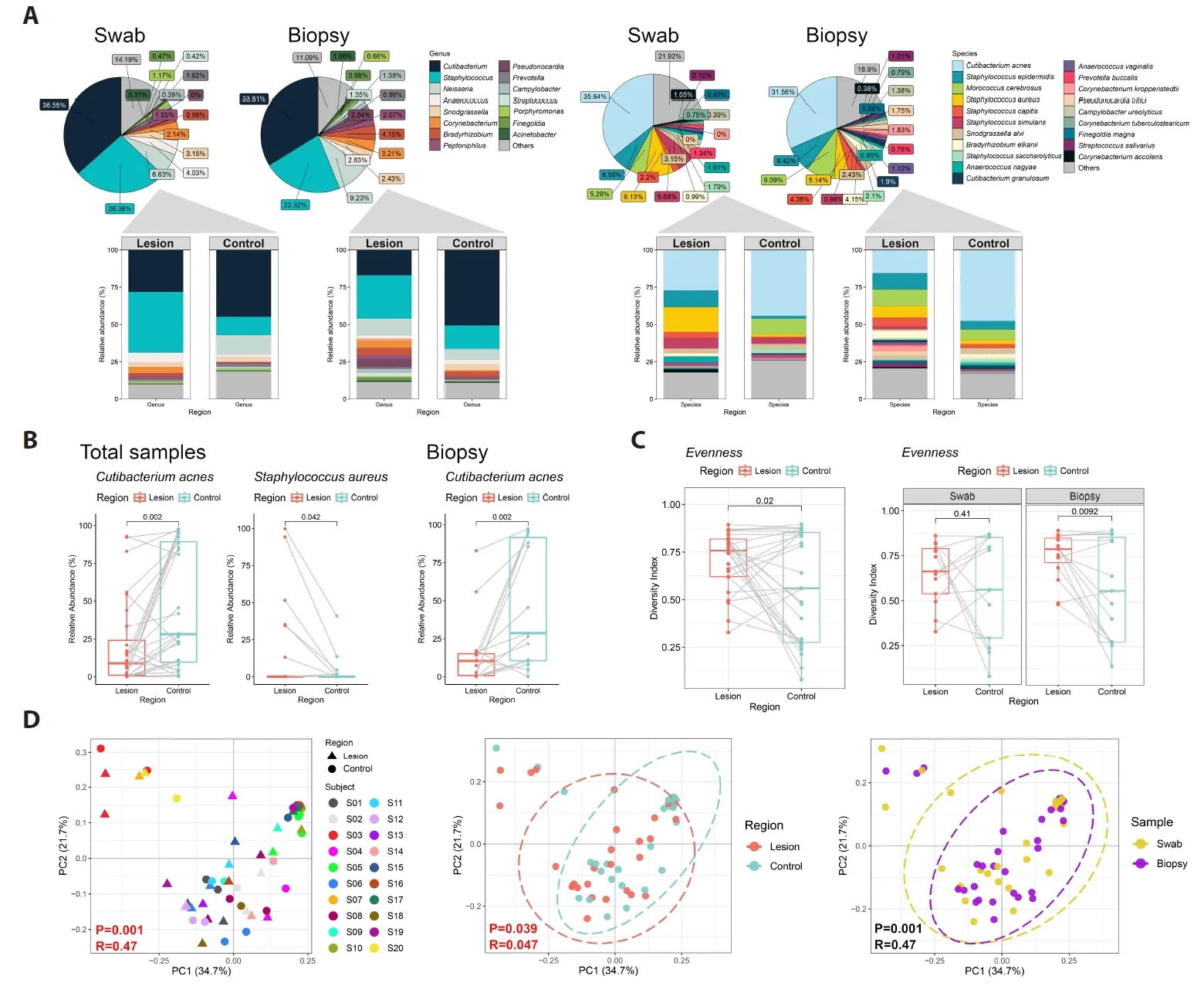
Skin microbiota profiling in basal cell carcinoma (BCC) among Korean patients (n = 20). (A) Relative abundance of the most prevalent bacterial genera and species in paired swab (n = 13 pairs) and biopsy (n = 15 pairs) samples. (B) Bacterial species showing differences in mean relative abundance between BCC lesions and contralateral clinically normal skin (Control, Ctrl) in the combined dataset (swab and biopsy) and the biopsy-only dataset. (C) Alpha diversity (Evenness) comparisons between BCC lesions and controls across the total dataset, biopsy dataset, and swab dataset. (D) Principal coordinates analysis (PCoA) based on weighted UniFrac distances, visualized by individual, sampling method, and skin site.

**Fig. 2.**
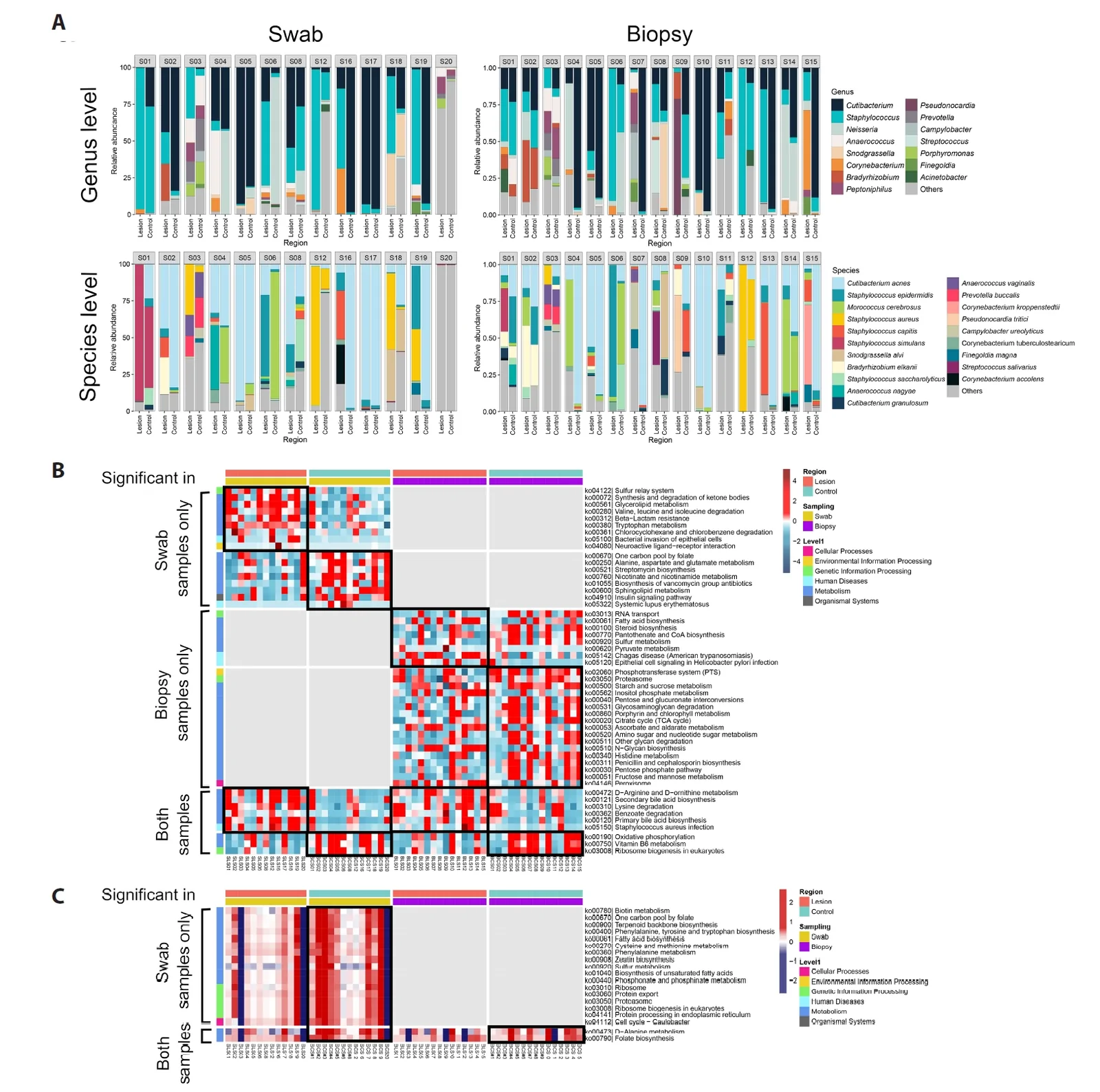
Microbiota composition and predicted functional profiles in BCC. (A) Relative abundance of the most prevalent bacterial genera and species in swab (n = 13 pairs) and biopsy (n = 15 pairs) datasets. (B) Predicted metagenomic functional pathways (PICRUSt2) showing the top differentially abundant KEGG pathways between BCC lesions and controls. (C) Comparison of *C. acnes*–associated KEGG pathway proportions between BCC lesions and controls.

Across all samples, sequencing identified 450 bacterial species spanning 243 genera, 28 classes, and 13 phyla. The dominant genera were *Cutibacterium* and *Staphylococcus* (Figs. 1A and 2A). At the species level, *C. acnes* was the most abundant taxon. In biopsy-derived datasets, *C. acnes* showed a trend toward lower relative abundance in BCC lesions compared with contralateral skin, while swab datasets showed a similar but less pronounced pattern (Fig. 1B).

Microbial evenness tended to be higher in lesions, whereas richness showed no consistent difference (Fig. 1C).

### Exploratory multivariate analyses

**Fig. 3.**
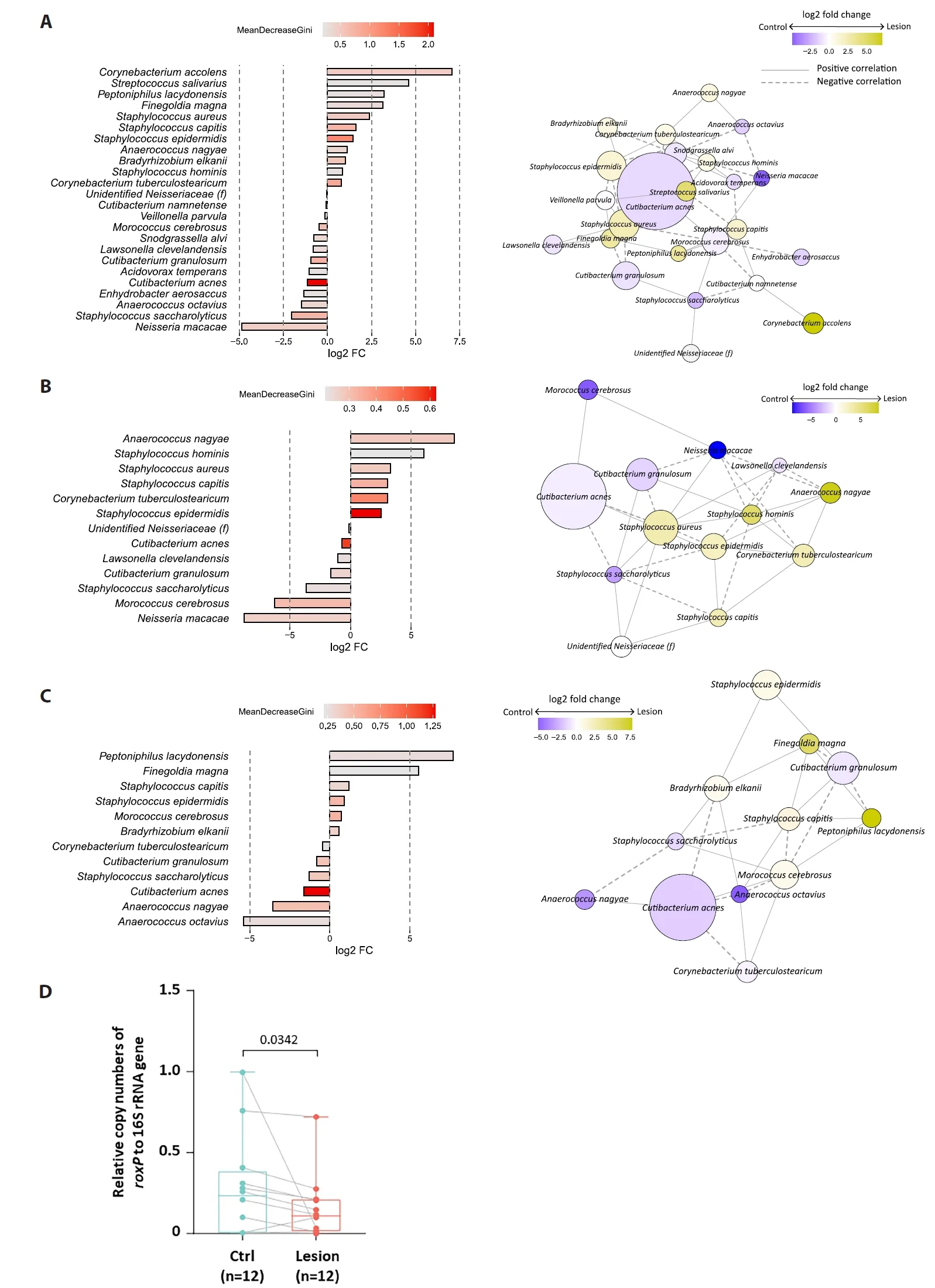
Discriminatory taxa and *roxP* antioxidant gene quantification in BCC. (A–C) Random forest analysis identifying the top 24 taxa (mean decrease Gini ≥ 0.2) distinguishing BCC lesions from controls in the total dataset (A), swab dataset (B), and biopsy dataset (C). Right panels: co-occurrence networks of amplicon sequence variants (ASVs) selected by random forest. Edges represent significant SparCC correlations (*P* < 0.05), with solid lines indicating positive and dashed lines indicating negative associations. Node size and color represent log₂ fold change between BCC and control. (D) Absolute quantification of the *roxP* gene (Radical oxygenase of *Propionibacterium acnes*) in swab samples (n = 12 pairs), expressed as the ratio of *roxP* to 16S rRNA copy number. Differences assessed by paired Wilcoxon signed-rank test; *P* < 0.05.

Beta diversity analyses showed partial clustering by individual, with modest separation between lesion and control samples (Fig. 1D). Random Forest analysis ranked *C. acnes* among the top taxa contributing to lesion–control differentiation (Fig. 3A). For swab samples, the model exhibited modest discrimination (AUC = 0.71) with low out-of-bag accuracy, whereas biopsy-based models demonstrated near-random performance (AUC = 0.56); accordingly, given the small sample size and high dimensionality, Random Forest analyses were treated as exploratory and interpreted as descriptive, serving primarily for pattern identification and feature ranking rather than predictive inference. SparCC network analysis suggested altered interspecies associations in lesion samples (Fig. 3A–3C), but these patterns require validation.

### Predicted functional profiles

PICRUSt2-based inference suggested lower representation of vitamin B6 metabolism pathways in BCC lesions and relatively higher folate biosynthesis in controls, particularly among *C. acnes*-associated ASVs (Fig. 2B–2C). These predictions are exploratory and hypothesis-generating.

### *roxP* gene quantification

In 12 paired swab samples, the *roxP*/16S ratio tended to be lower in BCC lesions than in controls, with substantial inter-individual variability (Fig. 3D). Given the limited sample size and potential confounding by overall *C. acnes* abundance, these results are interpreted cautiously.

## Discussion

This exploratory pilot study describes differences in skin bacterial community structure between BCC lesions and contralateral clinically normal skin in a Korean cohort. The most consistent trend was a relative reduction of *C. acnes* in lesion samples, particularly in biopsy-derived datasets, accompanied by increased microbial evenness. Similar community-level features—namely reduced dominance of sebaceous-associated taxa and increased evenness—have been reported in photodamaged skin and in keratinocyte cancers such as actinic keratosis and cutaneous squamous cell carcinoma (Burns et al., 2019; Grant et al., 2024; Voigt et al., 2022), although direct comparisons are limited by methodological differences.

Previous studies in predominantly Western populations have reported that chronic UV exposure and non-melanoma skin cancers are associated with shifts in cutaneous microbial communities, including reduced dominance of sebaceous-site commensals and increased community evenness or diversity in lesional or photodamaged skin (Patra et al., 2019; Woo et al., 2022). Microbiota studies in actinic keratosis and SCC further suggest that microbial composition may vary across the spectrum of UV-induced field cancerization (Krueger et al., 2022; Voigt et al., 2022; Wood et al., 2018), supporting the plausibility that BCC-associated lesions may also exhibit distinct microbial patterns. UV radiation has been shown to directly alter bacterial viability and metabolism, as well as indirectly shape microbial communities through effects on skin barrier function, immune responses, and oxidative stress (Nakatsuji et al., 2021; Patra et al., 2019). In this context, the observed reduction of *C. acnes* in BCC lesions in our study aligns with prior observations that sebaceous-associated taxa may be depleted in environments characterized by chronic inflammation, tissue remodeling, or tumor-associated microenvironmental changes. Importantly, data on skin microbiota in Asian patients with BCC remain scarce, and our findings extend existing literature by providing preliminary insights from a Korean cohort.

Methodological limitations substantially constrain interpretation. Biopsy sampling required lidocaine anesthesia, which may suppress bacterial viability. DNA extraction bias, prolonged frozen storage, low microbial biomass, and the absence of sequencing-based negative controls further limit quantitative inference. Anatomical site heterogeneity and systemic comorbidities may also influence observed microbiota patterns.

The observed reduction in *roxP* abundance likely reflects decreased *C. acnes* representation rather than reduced antioxidant capacity per bacterium. *C. acnes* encodes multiple oxidative stress defense systems, including catalases, peroxidases, and thioredoxin-related pathways, and *roxP* represents only one component of its broader redox biology. While RoxP has been shown to exert extracellular antioxidant activity in vitro (Andersson et al., 2019; Brüggemann et al., 2021), its relative contribution to oxidative balance on human skin in vivo remains incompletely defined. Accordingly, *roxP* should be viewed as a surrogate marker of community shifts rather than a dominant functional determinant.

Predictive functional differences observed in this study should be interpreted with particular caution. PICRUSt2 results represent inferred genomic potential rather than direct evidence of in vivo activity. Although reference genomes indicate that *C. acnes* harbors genes involved in folate and one-carbon metabolism, 16S-based inference cannot determine whether these pathways are transcriptionally active or biologically relevant within the BCC microenvironment. Future studies incorporating shotgun metagenomics and functional assays will be required.

## Conclusion

This pilot study provides preliminary evidence of altered skin microbiota composition in BCC lesions compared with contralateral skin in Korean patients. While trends suggest reduced *C. acnes* representation and altered inferred functional potential, substantial methodological limitations preclude causal inference. Larger, methodologically optimized studies are required to clarify the role of skin microbiota in BCC-associated microenvironmental changes.

## Figures and Tables

**Table 1.** Patient characteristics

Subject number	Sex/Age	Race	Diagnosis	Pathology	Pigment	Location	Swab	Biopsy	Co-morbidity
1	F/77	Korean	BCC	Nodulocystic	Present	Back	O	O	Peripheral neural disease
2	M/87	Korean	BCC	Superficial	Present	Back	O	O	DM
3	F/100	Korean	BCC	Nodulocystic	Present	Cheek	O	O	HTN
4	M/68	Korean	BCC	Nodulocystic	Present	Nose	O	O	HTN
5	M/53	Korean	BCC	Superficial	Present	Nose	O	O	None
6	F/82	Korean	BCC	Micronodular	Present	Cheek	O	O	Vitiligo
7	M/71	Korean	BCC	Nodulocystic	Present	Perinasal area	X	O	None
8	M/73	Korean	BCC	Nodulocystic	Present	Lower eyelid	O	O	DM, dyslipidemia
9	F/86	Korean	BCC	Nodulocystic	Present	Cheek	X	O	HTN
10	M/71	Korean	BCC	Nodulocystic	Present	Nose	X	O	Parkinson’s disease, cataract, HTN, DM
11	F/68	Korean	BCC	Superficial	Present	Back	X	O	HTN
12	F/80	Korean	BCC	Nodulocystic	Present	Upper eyelid	O	O	Stroke
13	M/81	Korean	BCC	Nodulocystic	Present	Nose	X	O	DM, HTN, CKD
14	M/62	Korean	BCC	Superficial	Present	Perioral area	X	O	None
15	M/82	Korean	BCC	Nodulocystic	Present	Perinasal area	X	O	HTN
16	F/97	Korean	BCC	Superficial	Present	Perioral area	O	X	DM, HTN, CKD
17	M/74	Korean	BCC	Micronodular	Present	Cheek	O	X	HTN
18	F/74	Korean	BCC	Nodulocystic	Absent	Nose	O	X	DM
19	F/79	Korean	BCC	Nodulocystic	Present	Lower eyelid	O	X	HTN, dyslipidemia
20	F/80	Korean	BCC	Superficial	Present	Perianal area	O	X	None

## Data Availability

Sequencing data will be deposited in the NCBI Sequence Read Archive (SRA) prior to publication; accession number(s) will be provided in the revised proof.
